# Vaccine fatigue and influenza vaccination trends across Pre-, Peri-, and Post-COVID-19 periods in the United States using epic’s cosmos database

**DOI:** 10.1371/journal.pone.0326098

**Published:** 2025-06-17

**Authors:** Tyler B. Nofzinger, Timothy T. Huang, Christopher Eduard R. Lingat, Gaurang M. Amonkar, Emily E. Edwards, Albert Yu, Alexander D. Smith, Nasser Gayed, Heidi L. Gaddey

**Affiliations:** 1 Carle Illinois College of Medicine, University of Illinois Urbana-Champaign, Champaign, Illinois, United States of America; 2 Carle Foundation Hospital, Urbana, Illinois, United States of America; 3 Department of Statistics, University of Illinois at Urbana-Champaign, Urbana, Illinois, United States of America; 4 Department of Computer Science, University of Illinois Urbana-Champaign, Champaign, Illinois, United States of America; Centers for Disease Control and Prevention, UNITED STATES OF AMERICA

## Abstract

**Introduction:**

Influenza vaccination is a critical public health measure, especially amidst the challenges of the COVID-19 pandemic. This study uses Epic’s Cosmos database to analyze influenza vaccination trends across demographic groups in the United States, examining the effects of the COVID-19 pandemic and “vaccine fatigue” on influenza vaccination rates.

**Methods:**

This retrospective cross-sectional study analyzes influenza vaccination rates pre- (1 May 2018–31 May 2019), peri- (1 May 2020–31 May 2021), and post-COVID-19 (1 May 2023–31 May 2024). A two-proportion z-test and Cohen’s h test were calculated to assess statistical significance.

**Results:**

Influenza vaccination rates increased peri-COVID (+1.99% point change, h = 0.04, p < 0.001) and decreased post-COVID (−6.41% point change, h = 0.14, p < 0.001) relative to pre-COVID. The largest changes were observed in the following groups: 5−18 year olds (−13.92% point change, h = 0.31, p < 0.001), 19−26 year olds (−9.91% point change, h = 0.25, p < 0.001), American Indian or Alaska Native (−8.11% point change, h = 0.18, p < 0.001), Other Races (−7.36% point change, h = 0.17, p < 0.001), White (−6.89% point change, h = 0.15, p < 0.001), and the South U.S. census region (−7.21% point change, h = 0.17, p < 0.001).

**Conclusion:**

Post-pandemic influenza vaccination compliance decreased relative to pre-pandemic, especially among younger age groups, certain racial groups, and in the southern U.S. These findings suggest that the COVID-19 pandemic negatively impacted influenza vaccination compliance. While overall trends aligned with publicly available data, absolute counts may be under-reported within Epic due to incomplete documentation of vaccine administrations outside of Epic-affiliated systems.

## Introduction

The coronavirus disease 2019 (COVID-19) pandemic, which was caused by severe acute respiratory syndrome coronavirus 2 (SARS-CoV-2), remains a major public health crisis and a polarizing political issue, with divisions arising over mask mandates, lockdowns, and vaccination policies [1,2,3]. This study aims to examine influenza vaccination compliance before, during, and after the COVID-19 pandemic to investigate the influence of “vaccine fatigue”, or the resultant disinterest in receiving vaccines or their related information due to a wide range of factors such as misconceptions regarding negative side effects, disease severity, and diminished trust in government and media [4]. While existing literature mostly relies on survey data, insurance claims, or census data to analyze general trends in influenza vaccination, this study leverages Epic’s Cosmos database to objectively examine influenza vaccination trends across diverse demographics within the United States (U.S.) [5,6,7,8].

Cosmos is a data set created in collaboration with a community of Epic health systems, representing more than 262 million unique patient records from 1,557 hospitals and over 35,500 clinics across all U.S. states and Lebanon as of 18 September 2024. This vast data set enables a detailed examination of vaccination trends before, during, and after the COVID-19 pandemic, offering insights into how the pandemic has influenced influenza vaccination compliance. This study shows this impact by stratifying patient influenza data by demographics such as age group, race, legal sex, and U.S. census region.

As nations responded to the pandemic, routine immunization programs, including those for influenza, faced several challenges [9]. Understanding these shifts in vaccination compliance is crucial for safeguarding public health. Moreover, analyzing vaccination trends in the context of the COVID-19 pandemic may provide insights into the factors influencing vaccine compliance and the effectiveness of public health interventions during a global health crisis [10].

Seasonal influenza remains a significant public health concern, causing substantial morbidity and mortality each year [11]. Additionally, the interplay between influenza, vaccine fatigue, and COVID-19 poses additional risks such as superinfections and outbreaks that are prone to generating strains on healthcare systems and subsequent negative patient outcomes [12]. According to a systematic review published in 2021 that included 6,639 articles on COVID-19 superinfection, the pooled prevalence of coinfection was 19% such that influenza type A and B were the most frequently reported viruses among patients who showed an increased mortality as a result [12]. As such, maintaining and improving influenza vaccination rates is of paramount importance to mitigate these risks.

This research addresses a critical gap in the current literature by directly establishing the connections between the COVID-19 pandemic, vaccine fatigue, and influenza vaccination rates across demographics. These findings aim to inform public health policies and interventions aimed at enhancing vaccination compliance and protecting public health amidst ongoing and future health crises.

## Methods

A retrospective cross-sectional analysis was performed using patient data queried from the Epic Cosmos database, a population health research tool developed by Epic Systems Corp. containing approximately 289 million patient records from the U.S. and Lebanon as of January 2025. Patients were categorized based on their influenza vaccination status as either having a recorded influenza vaccination or not within Epic during the following three distinct time periods:

- Pre-COVID-19: 1 May 2018–31 May 2019
- Peri-COVID-19: 1 May 2020–31 May 2021
- Post-COVID-19: 1 May 2023–31 May 2024

These time periods were selected in accordance with the first reported case of COVID-19 in the U.S. (19 January 2020) and the end of the federal COVID-19 public health emergency declaration (11 May 2023) [13,14]. The post-COVID-19 date range was intended to capture a transitional period from the end of the emergency phase into the post-COVID-19 era. This approach allows for the inclusion of data that reflects the immediate aftermath of the mandate's conclusion.

The study population was then divided into age groups in accordance with the U.S. Centers for Disease Control and Prevention (CDC) vaccine standard: 5−18, 19−26, 27−49, 50−64, and 65 + years old [15]. Demographic information was also collected, including legal sex, U.S. census region (West, Midwest, Northeast, and South), and race (Black or African American, White, Asian, American Indian or Alaska Native, Native Hawaiian or Other Pacific Islander, or Other), for each date range (see S2-S4 Tables for pre-, peri-, and post-COVID-19 vaccination rates, respectively). The following inclusion and exclusion criteria were uniformly applied across all three time periods:

**Inclusion Criteria:**

- Patient records with at least one encounter in the respective date range
- Patient records with at least two encounters in any two-year period

**Exclusion Criteria:**

- Patient records missing data for legal sex, age, race, or U.S. census region
- Patients under five years old, due to variable immunization schedules that could confound observed trends

Notably, missing data in certain demographic fields can lead to discrepancies in summative patient counts. Specifically, a patient may be excluded from a demographic group while remaining included in the overall count of all patients. However, all demographic groups are derived from the same base population defined by the all-patient variable count.

The data analysis included descriptive statistics for all patients, stratified by legal sex, U.S. census region, age, and race. Influenza vaccination rates were determined for each demographic group across the three time periods, defined as any influenza immunization type recorded directly within Epic. This includes immunizations administered during an encounter, historical administrations entered manually by clinicians, and records reported by external state registries or Care Everywhere exchanges – a platform within Epic that enables electronic sharing of health records across organizations to support continuity of care – within the specified date ranges. For patients with multiple influenza immunizations within a given date range, records were de-duplicated within Epic Cosmos so that that each patient was counted only once. All influenza vaccine types, listed in S1 Table, were included in this study.

### Statistical methods

Two tailed two-proportion Z-tests were manually calculated to compare proportions of influenza vaccine rates for all patients and specific demographic groups (legal sex, age, race, and U.S. census region) within the following date ranges: pre-COVID-19 vs. peri-COVID-19 and pre-COVID-19 vs. post-COVID-19. Significance α = 0.05 for all tests.

Given the large sample sizes, most tests were expected to be statistically significant. As such, percent changes for different groups were calculated to identify meaningful differences in influenza vaccination rates. Percent changes were quantified via an absolute percent difference and a Cohen’s h test [16], the latter of which was used to measure the effect size of proportional changes. Given the large sample size in this study, thresholds for small, medium, and large effect size were designated as h = 0.1, 0.4, and 0.7, respectively.

The study adhered to relevant ethical guidelines and regulations. Patient data accessed from the Cosmos database was anonymized to ensure privacy and confidentiality. By leveraging the extensive data available in Epic’s Cosmos database, this study provides a detailed and comprehensive analysis of influenza vaccination trends across various demographics in the context of the COVID-19 pandemic.

## Results

A total of 273,879,751 patients were recorded in Cosmos as of 18 September 2024. After applying inclusion and exclusion criteria for the pre-, peri-, and post-COVID-19 date ranges, 79,851,245 patients, 94,895,860 patients, and 103,232,583 patients remained for analysis, respectively.

**Fig 1:**
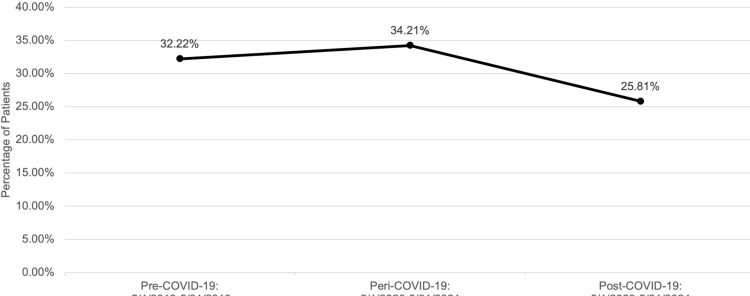
Influenza vaccination rates during pre-, peri-, and post-COVID-19 periods.

Influenza vaccination rates decreased from pre- to post-COVID-19 pandemic. Specifically, there was a 6.41% decrease (h = 0.14, p < 0.001) in reported influenza vaccination compliance for the total sample population when comparing the pre-COVID-19 and post-COVID-19 rates, which can be seen in Fig 1 (Note: all reported percent changes reflect percentage point changes). Fig 1 also shows that the influenza vaccination rate increased 1.99% (h = 0.04, p < 0.001) during the peri-COVID-19 period compared to the pre-COVID-19 period.

Influenza vaccination rates were then stratified by demographic subgroups across all three time periods. From the pre- to peri-COVID-19 periods, vaccination rates increased in most demographic subgroups. From the pre- to post-COVID-19 periods, influenza vaccination rates decreased in all demographic groups. See Tables 1 and 2 for influenza vaccination rates within specific demographic groups from the pre- to peri-COVID-19 and pre- to post-COVID-19, respectively.

**Table 1:** Influenza vaccine compliance, Pre- vs. Peri-COVID-19.

	Influenza Vaccine Pre-COVID-19 (%)	Influenza Vaccine Peri-COVID-19 (%)	Percent Point Change (Cohen’s h test)	p-value
**All Patients**	32.22%	34.21%	1.99% (h = 0.04)	< 0.001
**Age Groups (Years Old)**				
5-18	34.87%	37.14%	2.27% (h = 0.05)	< 0.001
19-26	24.14%	23.87%	−0.27% (h = 0.01)	< 0.001
27-49	21.63%	23.46%	1.84% (h = 0.04)	< 0.001
50-65	28.58%	32.95%	4.36% (h = 0.09)	< 0.001
65+	41.16%	46.02%	4.86% (h = 0.10)	< 0.001
**Legal Sex**				
Male	30.91%	32.39%	1.48% (h = 0.03)	< 0.001
Female	33.22%	35.64%	2.42% (h = 0.05)	< 0.001
**Race**				
American Indian or Alaska Native	31.41%	31.39%	−0.02% (h = 0.00)	< 0.001
Asian	36.68%	39.34%	2.66% (h = 0.05)	< 0.001
Black or African American	23.99%	24.97%	0.98% (h = 0.02)	< 0.001
Native Hawaiian or Other Pacific Islander	31.23%	32.61%	1.37% (h = 0.03)	< 0.001
Other Race	30.39%	31.80%	1.41% (h = 0.03)	< 0.001
White	34.75%	37.15%	2.39% (h = 0.05)	< 0.001
None of the Above	19.95%	22.22%	2.27% (h = 0.06)	< 0.001
**U.S. Census Region**				
South	29.34%	30.25%	0.91% (h = 0.02)	< 0.001
Midwest	35.35%	39.03%	3.68% (h = 0.08)	< 0.001
Northeast	32.72%	35.36%	2.64% (h = 0.06)	< 0.001
West	33.65%	34.38%	0.73% (h = 0.02)	< 0.001

**Table 2:** Influenza vaccine compliance, Pre- vs. Post-COVID-19.

	Influenza Vaccine Pre-COVID-19 (%)	Influenza Vaccine Post-COVID-19 (%)	Percent Point Change (Cohen’s h test)	p-value
**All Patients**	32.22%	25.81%	−6.41% (h = 0.14)	< 0.001
**Age Groups (Years Old)**				
5-18	34.87%	20.95%	−13.92% (h = 0.31)	< 0.001
19-26	24.14%	14.23%	−9.91% (h = 0.25)	< 0.001
27-49	21.63%	17.74%	−3.89% (h = 0.10)	< 0.001
50-65	28.58%	25.72%	−2.86% (h = 0.06)	< 0.001
65+	41.16%	39.45%	−1.71% (h = 0.03)	< 0.001
**Legal Sex**				
Male	30.91%	24.38%	−6.53% (h = 0.15)	< 0.001
Female	33.22%	26.88%	−6.33% (h = 0.14)	< 0.001
**Race**				
American Indian or Alaska Native	31.41%	23.30%	−8.11% (h = 0.18)	< 0.001
Asian	36.68%	33.64%	−3.03% (h = 0.06)	< 0.001
Black or African American	23.99%	19.37%	−4.62% (h = 0.11)	< 0.001
Native Hawaiian or Other Pacific Islander	31.23%	25.66%	−5.57% (h = 0.12)	< 0.001
Other Race	30.39%	23.03%	−7.36% (h = 0.17)	< 0.001
White	34.75%	27.77%	−6.98% (h = 0.15)	< 0.001
None of the Above	19.95%	17.25%	−2.69% (h = 0.07)	< 0.001
**U.S. Census Region**				
South	29.34%	22.13%	−7.21% (h = 0.17)	< 0.001
Midwest	35.35%	29.30%	−6.05% (h = 0.13)	< 0.001
Northeast	32.72%	27.48%	−5.24% (h = 0.11)	< 0.001
West	33.65%	27.64%	−6.01% (h = 0.13)	< 0.001

The largest observed decreases in influenza vaccination rates from pre- to post-COVID-19 occurred in patients aged 5−18 years old (−13.92%, h = 0.31, p < 0.001) and 19−26 years old (−9.91%, h = 0.25, p < 0.001); patients identifying as American Indian or Alaska Native (−8.11%, h = 0.18, p < 0.001), Other Race (−7.36%, h = 0.17, p < 0.001), and White (−6.89%, h = 0.15, p < 0.001); and patients residing in the southern U.S. census region (−7.21%, h = 0.17, p < 0.001). See Table 3 for the largest changes in influenza vaccination rates from pre- to post-COVID-19.

**Table 3:** Largest changes in influenza vaccine rates, Pre- vs. Post-COVID-19.

Demographic	Percentage Point Change	Cohen’s H Test	p-Value
5–18 years old	−13.92%	0.31	< 0.001
19–26 years old	−9.91%	0.25	< 0.001
American Indian or Alaska Native patients	−8.11%	0.18	< 0.001
Other Race	−7.36%	0.17	< 0.001
White patients	−6.89%	0.15	< 0.001
Patients from the South U.S. region	−7.21%	0.17	< 0.001

## Discussion

The results of this study suggest that the COVID-19 pandemic negatively impacted influenza vaccination compliance, as evidenced by a substantial decrease in influenza vaccination rates from the pre- to post-COVID-19 periods. While all demographic groups displayed an overall decrease in influenza vaccination rates, these declines disproportionately affected younger age groups, specific racial groups, and patients residing in the southern U.S. census region. The largest decreases from pre- to post-COVID-19 occurred in patients aged 5–18 years old (−13.92%, h = 0.31, p < 0.001) and 19–26 years old (−9.91%, h = 0.25, p < 0.001); patients identifying as American Indian or Alaska Native (−8.11%, h = 0.18, p < 0.001), Other Race (−7.36%, h = 0.17, p < 0.001), and White (−6.89%, h = 0.15, p < 0.001); and patients residing in the southern U.S. census region (−7.21%, h = 0.17, p < 0.001). The 5–18 year old patients are particularly notable, given the increased number of hospitalizations due to influenza complications in this age group from 2022–2023 [17]. Influenza also has a high transmission rate through younger populations, potentially leading to more severe complications and hospitalizations in more vulnerable individuals [18].

A possible explanation for the statistically significant changes in reported influenza vaccination trends brings us to the concept of vaccination fatigue, which likely increased over the course of the pandemic. The prolonged exposure to pandemic-related stressors such as mask mandates, lockdowns, and vaccination policies may have fostered passive or even resistant attitude toward vaccines, information about vaccines, or public safety [1,2,3,4]. Additionally, factors such as the lifting of public health mandates, a reduced sense of urgency around influenza vaccination after the pandemic, and ongoing politicization of vaccines likely exacerbated vaccine fatigue in the general population.

When comparing this study’s observed trends with publicly available CDC-reported estimates, both datasets showed an increase in influenza vaccination from pre- to peri-COVID-19 and a decrease in influenza vaccination from pre- to post-COVID-19 [19]. According to the CDC’s Flu Vaccination Coverage report for 2023−2024, influenza vaccination rates decreased by 8.3 percentage points in patients aged 6 months to 17 years old compared to pre-COVID-19 (2019−2020 season), and adult (18 + years old) influenza vaccination rates have steadily declined since the 2020−2021 season, which was characterized by a mild increase in influenza vaccinations during the start of the COVID-19 pandemic [19]. Similarly, Epic’s Cosmos data showed a decrease of 6.41 percentage points from pre- to post-COVID-19, reinforcing the consistency of observed trends.

While Epic’s Cosmos data showed vaccination rates lower than those reported by the CDC across all three time periods, this study has several limitations that may explain these discrepancies. Firstly, incomplete vaccine reporting and record-keeping by patients or healthcare professionals within Epic may lead to underestimates. Secondly, certain clinics using Epic may not be in-network for some patients’ insurance plans, leading them to seek out vaccinations at community pharmacies or other health systems that do not use Epic nor allow for the sharing of vaccination information to begin with. Thirdly, the analysis of this study used slightly different periods than those used by the CDC and included a narrower range of patients. The analysis of this study, however, focused on trends across time rather than distinct counts, so these effects are likely negligible. This is supported by the fact that the trends in our data align with those reported by the CDC.

Finally, while all patients listed in Cosmos as of 18 September 2024 were included, this number continues to grow over time as new organizations, departments, and clinics join the Cosmos database. Therefore, the raw count of patients with encounters likely increased over the course of the periods used in this study. This most likely did not affect the results due to the focus on longitudinal records (see exclusion criteria) and trends.

## Conclusions

Overall, this study utilized Cosmos to directly analyze the effects of the COVID-19 pandemic on influenza vaccination compliance. The data suggests that influenza vaccination compliance decreased after the pandemic period compared to before. The overall decrease can likely be attributed to vaccine fatigue that compounded on itself over the course of the pandemic. The post-pandemic decrease in influenza vaccination rates among the total sample population raises the concern of a growing public health risk, especially due to the significant morbidity and mortality of influenza. As such, public health messaging may need to shift focus toward combating vaccine fatigue and emphasizing the importance of routine vaccination schedules. Additionally, an added focus on promoting vaccination in younger age groups is of paramount importance due to a combination of the high impact of influenza and a significant decrease in influenza vaccination rates in this patient population.

On the clinical side, the dropping compliance rate likely means a more proactive outreach and educational approach is needed, as well as vaccination reminders and schedule strategy adjustments for providers and hospitals/clinics. Vaccines are critical for protecting oneself and others, and it’s important that healthcare professionals, governing bodies, and institutions evaluate their methods of disseminating information and promoting vaccination to combat the effects of vaccine fatigue on vaccination compliance. Future studies will build on the results of this paper by surveying for reasons behind lack of influenza vaccine compliance, potentially identifying the underlying causes of vaccine fatigue with more precision.

## Supporting information

S1 Table Summary of included influenza vaccine types. (DOCX)

S2 Table Influenza vaccine compliance, Pre-COVID-19. (DOCX)

S3 Table Influenza vaccine compliance, Peri-COVID-19. (DOCX)

S4 Table Influenza vaccine compliance, Post-COVID-19. (DOCX)

## References

[1] Synowiec A, Szczepański A, Barreto-Duran E, Lie LK, Pyrc K. Severe acute respiratory syndrome coronavirus 2 (SARS-CoV-2): a systemic infection. Clin Microbiol Rev. 2021;34(2):e00133-20. doi: 10.1128/CMR.00133-20 33441314 PMC7849242

[2] Bolsen T, Palm R. Politicization and COVID-19 vaccine resistance in the U.S. Prog Mol Biol Transl Sci. 2022;188(1):81–100. doi: 10.1016/bs.pmbts.2021.10.002 35168748 PMC8577882

[3] Albrecht D. Vaccination, politics and COVID-19 impacts. BMC Public Health. 2022;22(1):96. doi: 10.1186/s12889-021-12432-x 35031053 PMC8758893

[4] Su Z, Cheshmehzangi A, McDonnell D, da Veiga CP, Xiang Y-T. Mind the “Vaccine Fatigue”. Front Immunol. 2022;13:839433. doi: 10.3389/fimmu.2022.839433 35359948 PMC8960954

[5] Lin J, Li C, He W. Trends in influenza vaccine uptake before and during the COVID-19 pandemic in the USA. Public Health. 2023;225:291–8. doi: 10.1016/j.puhe.2023.10.028 37956641

[6] Naderalvojoud B, Shah ND, Mutanga JN, Belov A, Staiger R, Chen JH, et al. Trends in influenza vaccination rates among a medicaid population from 2016 to 2021. Vaccines (Basel). 2023;11(11):1712. doi: 10.3390/vaccines11111712 38006044 PMC10675465

[7] Parums DV. Editorial: global health concerns as vaccine-preventable infections including SARS-CoV-2 (JN.1), influenza, respiratory Syncytial Virus (RSV), and measles continue to rise. Med Sci Monit. 2024;30:e943911. doi: 10.12659/MSM.943911 38298093 PMC10845785

[8] Tarabichi Y, Frees A, Honeywell S, Huang C, Naidech AM, Moore JH, et al. The cosmos collaborative: a vendor-facilitated electronic health record data aggregation platform. ACI open. 2021;5(1):e36–46. doi: 10.1055/s-0041-1731004 35071993 PMC8775787

[9] Basu S, Ashok G, Debroy R, Ramaiah S, Livingstone P, Anbarasu A. Impact of the COVID-19 pandemic on routine vaccine landscape: a global perspective. Hum Vaccin Immunother. 2023;19(1):2199656. doi: 10.1080/21645515.2023.2199656 37078597 PMC10294763

[10] Cao Z, Yu R, Yuan Q, Ji W, Li X, Gao P, et al. Impact of the COVID-19 pandemic on routine vaccination coverage under varying prevalence Conditions: A cohort study in Beijing, China. Vaccine. 2024;42(2):213–9. doi: 10.1016/j.vaccine.2023.12.014 38097454

[11] World Health Organization. WHO, World Health Organization: WHO. Influenza (Seasonal). Published October 3, 2023. [cited September 2, 2024]. https://www.who.int/news-room/fact-sheets/detail/influenza-(seasonal)#:~:text=There%20are%20around%20a%20billion,650%20000%20respiratory%20deaths%20annually

[12] Musuuza JS, Watson L, Parmasad V, Putman-Buehler N, Christensen L, Safdar N. Prevalence and outcomes of co-infection and superinfection with SARS-CoV-2 and other pathogens: a systematic review and meta-analysis. PLoS One. 2021;16(5):e0251170. doi: 10.1371/journal.pone.0251170 33956882 PMC8101968

[13] Holshue ML, DeBolt C, Lindquist S, Lofy KH, Wiesman J, Bruce H, et al. First Case of 2019 novel coronavirus in the United States. N Engl J Med. 2020;382(10):929–36. doi: 10.1056/NEJMoa2001191 32004427 PMC7092802

[14] Laine C, Moyer DV. COVID-19 is no longer a public health emergency: implications for patients and clinicians. Ann Intern Med. 2023;176(7):983–4. doi: 10.7326/M23-1338 37216660 PMC10275403

[15] Vaccines by age. Vaccines & Immunizations. Published August 14, 2024. [cited September 2, 2024]. https://www.cdc.gov/vaccines/by-age/index.html

[16] Cohen J. Statistical power analysis for the behavioral sciences. 2nd ed. Hillsdale, NJ: Lawrence Erlbaum Associates. 1988.

[17] White EB, O’Halloran A, Sundaresan D, Gilmer M, Threlkel R, Colón A, et al. High influenza incidence and disease severity among children and adolescents aged <18 years - United States, 2022-23 Season. MMWR Morb Mortal Wkly Rep. 2023;72(41):1108–14. doi: 10.15585/mmwr.mm7241a2 37824430 PMC10578954

[18] Nayak J, Hoy G, Gordon A. Influenza in children. Cold Spring Harb Perspect Med. 2021;11(1):a038430. doi: 10.1101/cshperspect.a038430 31871228 PMC7778215

[19] Flu vaccination coverage, United States, 2023–24 influenza season. FluVaxView. Published September 20, 2024. [cited November 13, 2024]. https://www.cdc.gov/fluvaxview/coverage-by-season/2023-2024.html

