# Supplementary material for: Vaccine fatigue and influenza vaccination trends across Pre-, Peri-, and Post-COVID-19 periods in the United States using epic’s cosmos database
Source: PLoS One. 2025 Jun 17;20(6):e0326098. doi: 10.1371/journal.pone.0326098 (PMC12173228; doi:10.1371/journal.pone.0326098)
Supplement: S1 Table — (DOCX) [file pone.0326098.s001.docx]

**Supporting Information**

**S1 Table : Summary of included influenza vaccine types**

| **Influenza Vaccine Types (Total: 28)** |
| --- |
| Influenza Nasal, Unspecified (CVX 151)  Influenza, high dose seasonal (CVX 135)  Influenza, high dose, quadrivalent (CVX 197)  Influenza, injectable, MDCK, preservative free (CVX 153)  Influenza, injectable, MDCK, preservative free, quadrivalent (CVX 171)  Influenza, injectable, MDCK, quadrivalent, preservative (CVX 186)  Influenza, injectable, quadrivalent (CVX 158)  Influenza, Injectable, Quadrivalent, Preservative Free (CVX 150)  Influenza, Injectable, quadrivalent, Preservative Free, Pediatric (CVX 161)  Influenza, Intradermal, Quadrivalent, Preservative Free (CVX 166)  influenza, live, intranasal (CVX 111)  Influenza, live, intranasal, quadrivalent  influenza, live, intranasal, quadrivalent (CVX 149)  Influenza, recombinant, injectable, preservative free (CVX 155)  Influenza, recombinant, quadrivalent, injectable, preservative free (CVX 185)  Influenza, seasonal, injectable (CVX 141)  Influenza, seasonal, injectable, preservative free (CVX 140)  Influenza, seasonal, intradermal, preservative free (CVX 144)  Influenza, Seasonal, Quadrivalent, Adjuvanted (CVX 205)  Influenza, Southern Hemisphere (CVX 194)  influenza, Southern Hemisphere, high dose, quadrivalent (CVX 231)  Influenza, Southern Hemisphere, pediatric, preservative free (CVX 200)  Influenza, Southern Hemisphere, preservative free (CVX 201)  Influenza, Southern Hemisphere, quadrivalent, with preservative (CVX 202)  Influenza, Split (CVX 15)  Influenza, trivalent, adjuvanted (CVX 168)  Influenza, Unspecified (CVX 88)  Influenza, Whole (CVX 16) |
