# Supplementary material for: Vaccine fatigue and influenza vaccination trends across Pre-, Peri-, and Post-COVID-19 periods in the United States using epic’s cosmos database
Source: PLoS One. 2025 Jun 17;20(6):e0326098. doi: 10.1371/journal.pone.0326098 (PMC12173228; doi:10.1371/journal.pone.0326098)
Supplement: S2 Table — (DOCX) [file pone.0326098.s002.docx]

**Supporting Information**

**S2 Table: Influenza Vaccine Compliance, Pre-COVID-19**

|  | Influenza Vaccine Reported  (Distinct Count) | No Reported Influenza Vaccine  (Distinct Count) | Influenza Vaccine (%) |
| --- | --- | --- | --- |
| **All Patients** | 25,725,893 | 54,125,352 | 32.22% |
| **Age Groups (Years Old)** | | | |
| 5-18 | 3,852,936 | 7,196,330 | 34.87% |
| 19-26 | 1,822,548 | 5,726,799 | 24.14% |
| 27-49 | 4,380,077 | 15,874,089 | 21.63% |
| 50-65 | 4,707,691 | 11,761,638 | 28.58% |
| 65+ | 4,745,499 | 6,783,759 | 41.16% |
| **Legal Sex** | | | |
| Male | 10,682,313 | 23,878,386 | 30.91% |
| Female | 15,043,488 | 30,246,518 | 33.22% |
| **Race** | | | |
| American Indian or Alaska Native | 233,895 | 510,748 | 31.41% |
| Asian | 1,137,537 | 1,964,025 | 36.68% |
| Black or African American | 2,950,574 | 9,349,075 | 23.99% |
| Native Hawaiian or Other Pacific Islander | 128,171 | 282,187 | 31.23% |
| Other Race | 2,343,085 | 5,366,370 | 30.39% |
| White | 20,463,286 | 38,419,687 | 34.75% |
| None of the above | 616,710 | 2,474,812 | 19.95% |
| **U.S. Census Region** | | | |
| South | 8,745,435 | 21,064,826 | 29.34% |
| Midwest | 8,037,473 | 14,696,724 | 35.35% |
| Northeast | 5,478,356 | 11,265,340 | 32.72% |
| West | 3,433,973 | 6,772,024 | 33.65% |
