# Supplementary material for: Vaccine fatigue and influenza vaccination trends across Pre-, Peri-, and Post-COVID-19 periods in the United States using epic’s cosmos database
Source: PLoS One. 2025 Jun 17;20(6):e0326098. doi: 10.1371/journal.pone.0326098 (PMC12173228; doi:10.1371/journal.pone.0326098)
Supplement: S3 Table — (DOCX) [file pone.0326098.s003.docx]

**Supporting Information**

**S3 Table: Influenza Vaccine Compliance, Peri-COVID-19**

|  | Influenza Vaccine Reported  (Distinct Count) | No Reported Influenza Vaccine  (Distinct Count) | Influenza Vaccine (%) |
| --- | --- | --- | --- |
| **All Patients** | 32,468,037 | 62,427,823 | 34.21% |
| **Age Groups (Years Old)** | | | |
| 5-18 | 4,116,458 | 6,967,750 | 37.14% |
| 19-26 | 2,268,269 | 7,234,463 | 23.87% |
| 27-49 | 6,174,792 | 20,141,263 | 23.46% |
| 50-65 | 6,639,405 | 13,512,433 | 32.95% |
| 65+ | 6,295,433 | 7,383,124 | 46.02% |
| **Legal Sex** | | | |
| Male | 13,482,086 | 28,139,090 | 32.39% |
| Female | 18,985,854 | 34,288,319 | 35.64% |
| **Race** | | | |
| American Indian or Alaska Native | 289,877 | 633,614 | 31.39% |
| Asian | 1,615,182 | 2,490,612 | 39.34% |
| Black or African American | 3,539,472 | 10,636,350 | 24.97% |
| Native Hawaiian or Other Pacific Islander | 161,205 | 333,206 | 32.61% |
| Other Race | 3,057,374 | 6,557,512 | 31.80% |
| White | 25,512,361 | 43,170,659 | 37.15% |
| None of the above | 1,048,484 | 3,670,927 | 22.22% |
| **U.S. Census Region** | | | |
| South | 10,501,662 | 24,216,886 | 30.25% |
| Midwest | 9,924,835 | 15,502,215 | 39.03% |
| Northeast | 7,060,855 | 12,908,887 | 35.36% |
| West | 4,941,659 | 9,431,877 | 34.38% |
