# Supplementary material for: Vaccine fatigue and influenza vaccination trends across Pre-, Peri-, and Post-COVID-19 periods in the United States using epic’s cosmos database
Source: PLoS One. 2025 Jun 17;20(6):e0326098. doi: 10.1371/journal.pone.0326098 (PMC12173228; doi:10.1371/journal.pone.0326098)
Supplement: S4 Table — (DOCX) [file pone.0326098.s004.docx]

**Supporting Information**

**S4 Table: Influenza Vaccine Compliance, Post-COVID-19**

|  | Influenza Vaccine Reported  (Distinct Count) | No Reported Influenza Vaccine  (Distinct Count) | Influenza Vaccine (%) |
| --- | --- | --- | --- |
| **All Patients** | 26,640,599 | 76,591,984 | 25.81% |
| **Age Groups (Years Old)** | | | |
| 5-18 | 2,920,461 | 11,021,786 | 20.95% |
| 19-26 | 1,479,752 | 8,917,073 | 14.23% |
| 27-49 | 5,126,658 | 23,772,257 | 17.74% |
| 50-65 | 5,649,670 | 16,312,620 | 25.72% |
| 65+ | 5,771,950 | 8,858,999 | 39.45% |
| **Legal Sex** | | | |
| Male | 10,821,005 | 33,569,465 | 24.38% |
| Female | 15,819,526 | 43,022,169 | 26.88% |
| **Race** | | | |
| American Indian or Alaska Native | 251,263 | 827,204 | 23.30% |
| Asian | 1,466,168 | 2,891,902 | 33.64% |
| Black or African American | 3,072,780 | 12,789,131 | 19.37% |
| Native Hawaiian or Other Pacific Islander | 138,455 | 401,068 | 25.66% |
| Other Race | 2,611,357 | 8,725,549 | 23.03% |
| White | 20,502,707 | 53,322,866 | 27.77% |
| None of the above | 925,178 | 4,436,848 | 17.25% |
| **U.S. Census Region** | | | |
| South | 8,975,884 | 31,583,706 | 22.13% |
| Midwest | 7,903,489 | 19,066,813 | 29.30% |
| Northeast | 5,697,563 | 15,033,926 | 27.48% |
| West | 4,030,866 | 10,553,597 | 27.64% |
